# An open chat with…Alexander Wlodawer

**DOI:** 10.1002/2211-5463.13462

**Published:** 2022-07-07

**Authors:** Ioannis Tsagakis, Alexander Wlodawer

**Affiliations:** ^1^ FEBS Open Bio Editorial Office Cambridge UK; ^2^ Center for Structural Biology National Cancer Institute Frederick MD USA

## Abstract

Alexander Wlodawer has been a member of the *FEBS Open Bio* Editorial Board since the journal's launch in 2011. Currently, he is Senior Investigator at the Center for Structural Biology, National Cancer Institute in Frederick, Maryland, USA. He received his Ph.D. from the University of California, Los Angeles in 1974, completed postdoctoral training at Stanford University and has also worked at the National Bureau of Standards, the ABL‐Basic Research Program at the NCI‐FCRDC and the University of Cambridge, UK. He is Doctor *Honoris Causa* of the Technical University of Lodz, Poland. Alexander Wlodawer is also a recipient of the 2006 NCI Mentor of Merit Award, was awarded the Heyrovsky Honorary Medal by the Czech Academy of Sciences in 2008, was elected Foreign Member of the Polish Academy of Sciences in 2005, and has been member of the Editorial Board of *The FEBS Journal* since 2007. He is currently Editor‐in‐Chief of the journal *Current Research in Structural Biology*. In this compelling interview, he shares with us his experiences on solving the structures of IL‐4 and retroviral proteases, advice on how to deal with being scooped, and his thoughts on open data sharing and AlphaFold.

## Graphical abstract

Alexander Wlodawer is Senior Investigator at the Center for Structural Biology, National Cancer Institute in Frederick, Maryland, USA, and one of the inaugural members of the *FEBS Open Bio* Editorial Board. In this compelling interview, he shares with us his experiences on solving the structures of IL‐4 and retroviral proteases and his thoughts on open data sharing and AlphaFold.

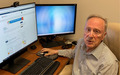

## As an expert in structural biology, what is your opinion on the role of this field in addressing the COVID‐19 pandemic, and how can this knowledge be used for vaccine design?

In a sense, it was exceedingly important; my daughter works in a company that is quite involved in these problems and she always keeps correcting me by saying that structural biology *per se* did not directly lead to the first vaccines. I don't necessarily agree with her completely, but you have to listen to your children sometimes! I think that at least for the inhibitors of the main protease of SARS‐CoV‐2, structural biology was crucial. I think all structures of the spike protein with different antibodies, although perhaps not important for the design of the first vaccines, will be important for future vaccines. Also, what turned out to be crucial were the new developments in structural biology, especially the introduction of cryo‐EM, as most COVID‐related structures were obtained using this technique. Most of that structural work would not have been possible a decade ago when cryo‐EM was still a fairly low‐resolution technology.

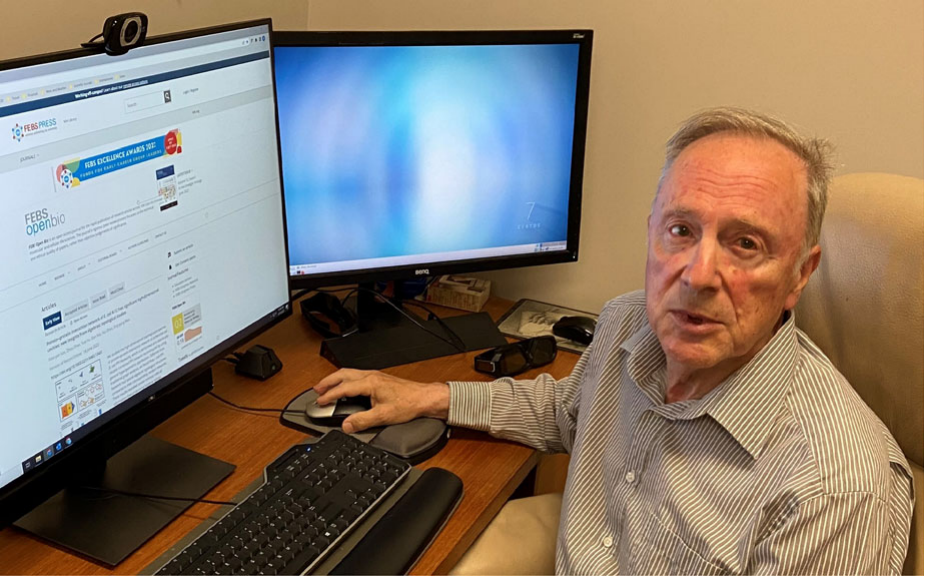

## If AlphaFold had been available earlier during the pandemic, do you think this would have helped accelerate vaccine development?

I think that AlphaFold may help, for example, in things like getting structures for molecular replacement, where you can verify the model and you get a better starting model from AlphaFold than any other models. So, it may be helpful to some, but it may not be helpful to others. I'm not trying to downplay its importance, [as] for the very big picture, for trying to think about those gigantic complexes where you have so many pieces, it may be extremely important.

## If not science, what career would you have pursued?

I don't know, I know of no other career… I was groomed to be a scientist since I was in kindergarten. I grew up among scientists, my mother was a professor of biochemistry and there were always scientists around. When I was, I guess 4 years old, I applied for a job in the institute where she worked. I went to the director of the institute and told him that I wanted to go there one day and ultimately I did, although only for a couple of months. So no, I don't know of any other career that I could have had.

## Nowadays it's not rare for young scientists to move countries to pursue a career in research. How commonplace was this when you moved to the United States and how would you describe your personal experience moving there?

I made a decision to move to the United States long before I really started science because I visited the United States in 1962, as a 16‐year‐old going to a meeting organized by the Junior Red Cross. That was a very important moment in my life. I spent 5 weeks there and I decided that as soon as I finish high school and college, I'm going to go to a US graduate school. I worked toward that particular goal and because of some complicated political problems in Poland, in some respects, it made [my move to the US] easier.

So at the time, it was not common at all, at least for people from Poland to go to the United States, but I was one of those lucky ones who could do that. Things changed later and many people from Europe came to do either undergraduate or graduate work in the United States and many post‐docs from Europe came to the United States. Only more recently, maybe in the last decade, the trend has changed and the United States, for a variety of reasons, became a much less desirable place for people, which is, of course, very sad for me but that's the case.

## Back then, was the performance of students and post‐docs assessed differently compared to nowadays?

I have had reasonably little experience with students because I [mostly] worked in government laboratories that do not really [host] students. But for post‐docs, of course, everything in the last few years became a matter of publishing in top journals. The journal became more important, in some respects, than the contents of their publications and unless people had all of these fantastic publications [in these] fantastic journals, it became much more difficult to get permanent positions.

## Have you ever had someone else try to scoop your findings?

Oh, of course, that happened more than once. Quite a few projects that we undertook were quite competitive and at least in one case it turned out that there were four groups trying to get the same IL‐4 structure. So, at the very end, what we did was to get all four groups together to write a fifth paper, comparing all these structures. I think that it's a reasonably valuable paper because these were comparisons of NMR and X‐ray structures of the same target, providing a different level of detail. In the end, I think being scooped is not that terrible, as long as you can still get something valuable from your data. I spend much time looking at structures from other laboratories, looking for problems. If we found any, we always tried to get the original authors to resubmit structures together with us or just by themselves, rather than just trying to say, gotcha, we found errors in your structure. So, that's how we try to do it, since I've been in this field for half a century …or maybe I am not as competitive as I was when I was much younger, so I don't mind.

## What was one of the most high‐risk, high‐reward projects you have ever undertaken and how did you manage the risks?

Clearly the highest risk project was [solving the structure of] retroviral proteases, particularly from HIV, because that was something which was directly related to a health crisis and I knew that there are lots of other people working on it. However, what helped in this case was that I was not in a grant‐supported institution. I had my internal budget and I could make a decision to just get into a field that was extremely competitive. The other [fields] were [for example] cytokine research, where there was also lots of competition but then there were lots of cytokines.

So, even though you would not necessarily be the first one to work on something, you could still do quite good work. Although it's much harder if it's all just grant‐supported. I hear stories about granting agencies that want people to repay the grants if they haven't published the results within their three‐year grant period and so on. … I am very grateful that my fate always put me in institutions where at least [there was] support given for long‐term projects.

## How did the experience of solving the structure of retroviral proteases derived from the Rous sarcoma virus and making the data freely available prior to publication change the way you approach scientific publishing?

… In that particular case, the race was really on, and it was a race with death because in the early days of the HIV pandemic, AIDS was a delayed death sentence. So, we were very much in favor of making data available immediately. Also, we felt that it was absolutely crucial to have the best possible model available given that people from an American pharmaceutical company published the structure of HIV protease, where there were some problems with their structure, without really giving any details.

Of course right now, the situation has changed by the presence of archives, like bioRxiv which has changed the mode of publication, not necessarily in the best possible way. I have seen cases where papers were published in bioRxiv but were later abandoned probably because they could not get them published in [high] impact factor journals.

In a sense, I'm not sure that is the best possible way [forward] and my approach, right now, is that I do not send my papers to bioRxiv. I prefer to have them peer evaluated before they are published as the lack of peer evaluation in those preliminary publications may hurt. On the other hand, for the current COVID pandemic, it's not papers but data that are released immediately. That was exceedingly important and really helped in the design of drugs that are available right now, and [this] probably helped a lot in the vaccine field as well.

## Sometimes we all mess up—what would you say is your biggest lab mistake [that you are willing to share, anyway]?

Nothing that I think was really very big, but yes, we did have cases where 10 years later we had to, for example, correct some structures that we deposited in the PDB because we found some very stupid errors that I absolutely do not understand how we allowed to slip in. But once we discovered them, we certainly decided that it's much better to correct them than to leave something in databases that is questionable.

## Have you learned anything different from acting as a mentor versus being the mentee?

Oh of course, I learnt that you have to very much listen to your mentees, of what they are trying to tell you because they can find things that you are not doing right, and I have been corrected by my mentees more than once. And it's always a very good experience when you interact with younger people who have different views on certain things and they convince you that they are right and you are not.

## What is it about scientists and climbing? Is it that climbing helps shape your work ethos in science, or training in science helps to equip you with skills that are critical in becoming a competitive climber?

That I don't know, to tell you the truth… [For me] it all started when I was still an undergraduate student at the physics department [where] quite a lot of people who were in my department were mountain climbing. My passion for climbing may also have something to do with my family history because my mother was a mountain climber in her young age, which was before World War Two, when it was not very common, for women especially. She started taking me to the mountains when I was 3 or 4 years old, so I just couldn't avoid it. I did real mountain climbing until the age of about 30 and I learned from a description of how various mountain climbers died in accidents, that if you're over 30, you either have to do it really seriously or stop because if you do it part‐time, you're going to be in real trouble. So I stopped, but I do go to the mountains as often as I can.

